# Synthesis of reactive 1,3-diphenyl-6-aryl-substituted fulvene chromophores

**DOI:** 10.1016/j.dib.2018.06.026

**Published:** 2018-06-22

**Authors:** Stephen M. Budy, David Y. Son, Gary J. Balaich, Scott T. Iacono

**Affiliations:** a Department of Chemistry, Southern Methodist University, Dallas, TX 75275, USA; b Department of Chemistry, Chemistry Research Center, United States Air Force Academy, Colorado Springs, CO 80919, USA

## Abstract

This data article describes a detailed synthetic strategy and experimental data for the synthesis of brominated fulvene chromophores as reactive precursors/monomers for conjugated organic materials. Metal-mediated coupling reactions of brominated fulvenes would result in conjugated small molecules or polymers that would find application as organic light emitting diodes (OLEDs) and photovoltaic (PV) applications.

**Specifications Table**

**Table:** 

Subject area	*Chemistry*
More specific subject area	*Synthesis of fulvene precursors*
Type of data	*Synthetic schemes, experimental synthesis protocols, NMR, FTIR, and MS results*
How data was acquired	*NMR: 400 MHz, solvent=CDCl*_*3*_*(Agilent); FTIR: Attenuated total reflectance Fourier transform infrared (Thermo Nicolet-FTIR Spectrometer iS10), MS: G1969 ESI-LC/MS-TOF (Agilent) or 7890A GC/5975C EI MS (Agilent)*
Data format	*Analyzed*
Experimental factors	*Starting compounds were either purchased, or synthesized using already published synthetic strategies*
Experimental features	*Compounds were synthesized and their structures were identified by NMR, FTIR, and MS*
Data source location	*Colorado Springs, CO and Dallas, TX USA*
Data accessibility	*Data are provided with this article*

**Value of the data**

• The data points to the synthesis of the aforementioned compounds.
• The brominated fulvenes could be used as intermediates for synthesizing conjugated small molecules or polymers using metal-mediated reactions.
• The conjugated small molecules and polyfulvenes could be used for organic light emitting diodes (OLEDs) and photovoltaic (PV) applications.

## 1 Data

All ^1^H and ^13^C NMR spectra were obtained using an Agilent Technologies 400 MHz instrument, and chemical shifts were reported in parts per million (*δ*) internally referenced to CDCl_3_ (^1^H NMR: *δ*=7.26 ppm and ^13^C NMR: *δ*=77.0 ppm). Attenuated total reflectance Fourier transform infrared (ATR-FTIR) spectra were collected using a Thermo Nicolet FTIR spectrometer iS10. Mass spectrometry results were obtained using an Agilent Technologies G1969 ESI-LC/MS-TOF instrument consisting of a gradient LC system coupled to a time-of-flight mass spectrometer and electrospray ionization source or an Agilent Technologies 7890A GC system interfaced with a 5975C EI mass spectrometer.

## 2 Experimental design, materials and methods

### 2.1 Materials

Solvents, starting materials, and reagents were purchased either from Sigma-Aldrich, TCI America, or Alfa Aesar as reagent grade or higher quality and used as received unless otherwise noted. HPLC grade THF was dried and deoxygenated by passage through Innovative Technologies Pure-Solv solvent purification system equipped with Cu/Al columns. Premium grade silica gel used for column chromatography was purchased from Sorbent Technologies (60 Å, 40−63 nm (230×400 Mesh)). All reactions and solvent transfers were carried out under an atmosphere of argon unless otherwise noted. All glassware was oven-dried prior to use.

### 2.2 Synthesis and characterization of compounds

#### 2.2.1 Synthesis of (1)

**Figure:**
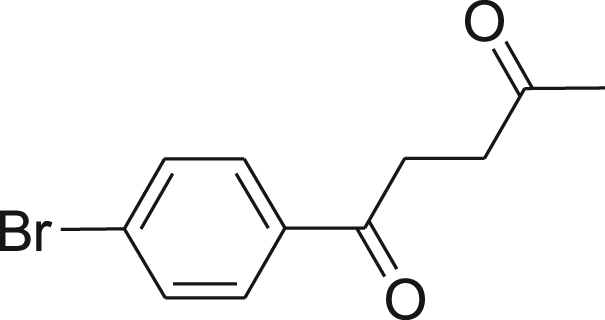

This compound was synthesized according to Ref. [1].

#### 2.2.2 Synthesis of (2)

**Figure:**
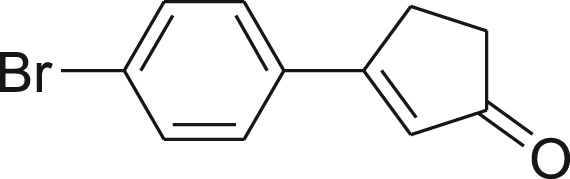

This compound was synthesized according to Ref. [1].

#### 2.2.3 Synthesis of (3)

**Figure:**
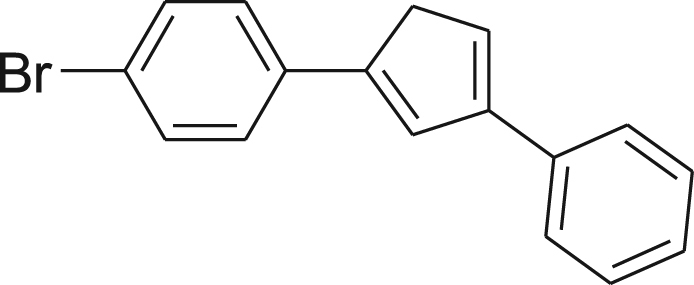

A prepared solution of phenylmagnesium bromide in THF (2 M, 19 mL, 38 mmol) under N_2_ was added dropwise over 30 min to a vigorously stirred solution of **2** (6.0 g, 25 mmol) in THF (200 mL) under N_2_. The resulting brown reaction mixture was allowed to stir at room temperature for 24 h under N_2_ and followed by GC–MS to ensure complete conversion. The reaction mixture was cooled in an ice bath with vigorous stirring and quenched with the slow addition of cold water (100 mL). Dropwise addition of H_2_SO_4_ (20 mL, 6 M) with vigorous stirring resulted in a brown organic layer and a clear aqueous layer. THF was evaporated under vacuum and the product was extracted with Et_2_O. The brown Et_2_O layer was separated from the acidic aqueous layer and washed sequentially with saturated NaHCO_3_ (3×200 mL), water (2×200 mL), and saturated brine (1×200 mL). The organic layer was dried over anhydrous MgSO_4_, filtered, and concentrated under vacuum to afford a brown solid and was triturated from acetone. Vacuum filtration, washing with cold acetone, and drying under reduced pressure afforded the product as a tan solid (3.96 g, 53%). ^1^H NMR (400 MHz, CDCl_3_): *δ* 3.76 (s, 2H), 6.95 (d, *J*=2.74 Hz, 2H), 7.24 (d, *J*=7.43 Hz, 1H), 7.36 (t, *J*=7.63 Hz, 2H), 7.39–7.49 (m, 4H), 7.56 (d, *J*=7.43 Hz, 2H); ^13^C NMR (100 MHz, CDCl_3_): *δ* 40.76, 124.89, 126.34, 126.98, 128.09, 128.68, 128.91, 131.71; MS (EI, 70 eV): *m*/*z* (% rel. int.) 298 (M^+^, 97), 296 (100), 215 (57), 202 (46), 189 (11), 139 (8), 115 (12), 107 (7), 94 (6).

#### 2.2.4 Synthesis of (4)

**Figure:**
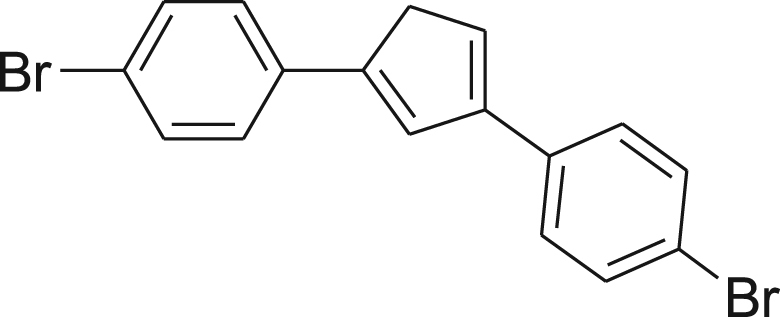

A freshly prepared solution of 4-bromophenylmagnesium bromide in Et_2_O (40 mL, 12.7 mmol) at 0 °C was added dropwise over 30 min to a vigorously stirred solution of **2** (2.0 g, 8.4 mmol) in Et_2_O (200 mL) under N_2_. The resulting brown reaction mixture was allowed to stir at room temperature for 24 h under N_2_ followed by GC–MS to ensure complete conversion. Additional reflux up to 24 h and addition of freshly prepared Grignard reagent was often necessary to achieve conversion. The reaction mixture was cooled in an ice bath and quenched with the slow addition of cold water (100 mL). Dropwise addition of H_2_SO_4_ (10 mL, 6 M) with vigorous stirring resulted in a brown Et_2_O layer and a clear water layer, which was stirred for an additional 5 min. The brown Et_2_O layer was separated from the acidic aqueous layer and washed sequentially with saturated NaHCO_3_ (3×200 mL), water (2×200 mL), and saturated brine (1×200 mL). The organic layer was dried over anhydrous MgSO_4_, filtered, and concentrated under vacuum to afford a brown solid and triturated in hexane/ethyl acetate (70:30). Vacuum filtration, washing with cold hexane, and drying under reduced pressure afforded the product as a tan solid (1.67 g, 53%). ^1^H NMR (400 MHz, CDCl_3_): *δ* 3.72 (s, 2H), 6.94 (s, 2H), 7.38–7.50 (m, 8H); ^13^C NMR (100 MHz, CDCl_3_): *δ* 34.91, 115.71, 121.45, 123.43, 127.63, 131.91, 137.25, 144.69; HRMS (ESI): Calcd for [M + H]^+^, 374.9386, Found, 374.9377.

#### 2.2.5 Synthesis of (5)

**Figure:**
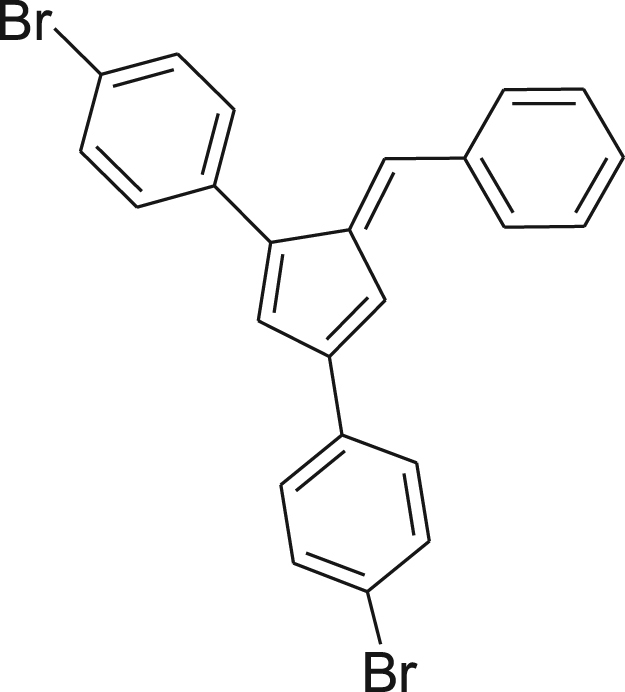

Pyrrolidine (0.57 g, 0.67 mL, 8.0 mmol) was added drop wise to a vigorously stirred slurry of benzaldehyde (0.68 g, 0.65 mL, 6.4 mmol) and **4** (2.0 g, 5.3 mmol) in absolute EtOH (200 mL). The reaction mixture was stirred for 24 h at room temperature. The red mixture was concentrated by evaporating the solvent to half the volume, vacuum filtered, washed with cold EtOH, and dried under reduced pressure affording the pure product as a dark red solid (2.23 g, 90%). ^1^H NMR (400 MHz, CDCl_3_): *δ* 6.95 (d, *J*=1.57 Hz, 1H), 7.03–7.07 (m, 1H), 7.26 (s, 1H) 7.31–7.36 (m, 2H), 7.39–7.61 (m, 9H), 7.63 (d, *J*=7.04 Hz, 2H); ^13^C NMR (100 MHz, CDCl_3_): *δ* 115.27, 121.33, 121.90, 127.57, 127.81, 128.82, 129.40, 130.67, 130.85, 131.57, 131.80, 133.89, 134.60, 136.68, 139.16, 140.55, 143.79, 145.51; HRMS (ESI): Calcd for [M+H]^+^, 464.9478, Found, 464.9358.

#### 2.2.6 Synthesis of (6)

**Figure:**
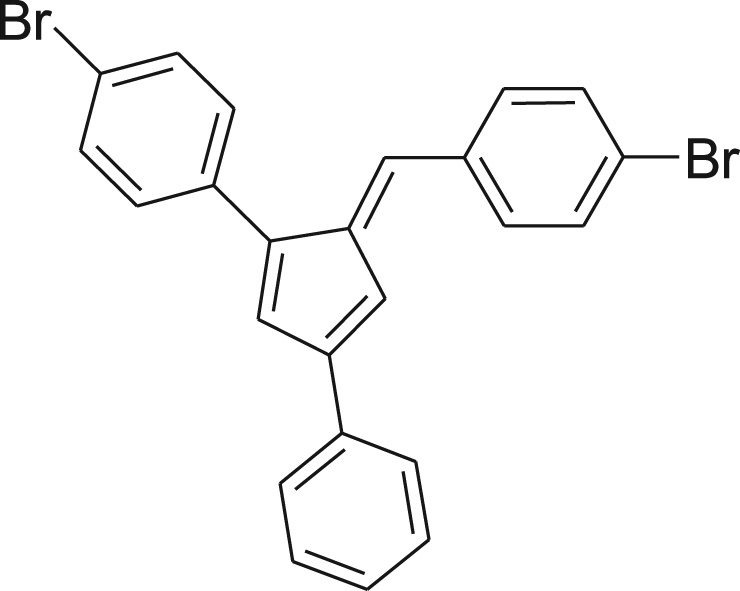

Pyrrolidine (1.8 g, 2.1 mL, 25.2 mmol) was added drop wise to a vigorously stirred slurry of 4-bromobenzaldehyde (3.7 g, 20.2 mmol) and **3** (5.0 g, 16.8 mmol) in absolute EtOH (200 mL). The reaction mixture was stirred for 24 h at room temperature. The red mixture was concentrated by evaporating the solvent to half volume, vacuum filtered, washed with cold EtOH, and dried under reduced pressure affording the pure product as a dark red solid (5.6 g, 72%). ^1^H NMR (400 MHz, CDCl_3_): *δ* 6.94–7.02 (m, 2H), 7.12 (s, 1H), 7.21 (s, 1H), 7.31–7.36 (m, 2H), 7.37–7.61 (m, 10H), 7.68–7.72 (m, 1H); ^13^C NMR (100 MHz, CDCl_3_): *δ* 114.25, 121.33, 122.00, 123.72, 126.11, 127.61, 128.25, 128.47, 128.72, 129.32, 130.83, 131.83, 131.99, 133.90, 134.77, 135.69, 136.76, 140.34, 144.52, 146.01, 147.22; HRMS (ESI): Calcd for [M+H]^+^, 464.9478, Found, 464.9458.

#### 2.2.7 Synthesis of (7)

**Figure:**
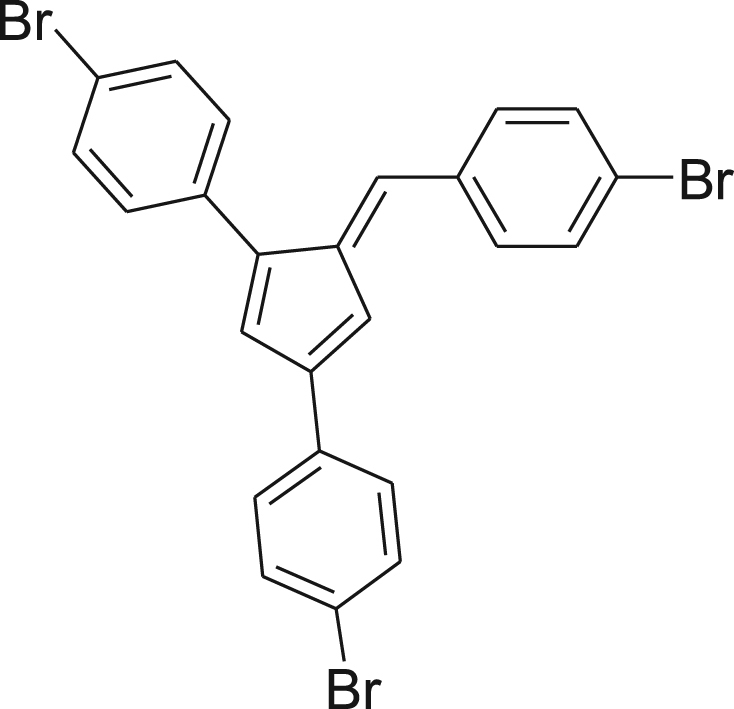

**Scheme 1:**
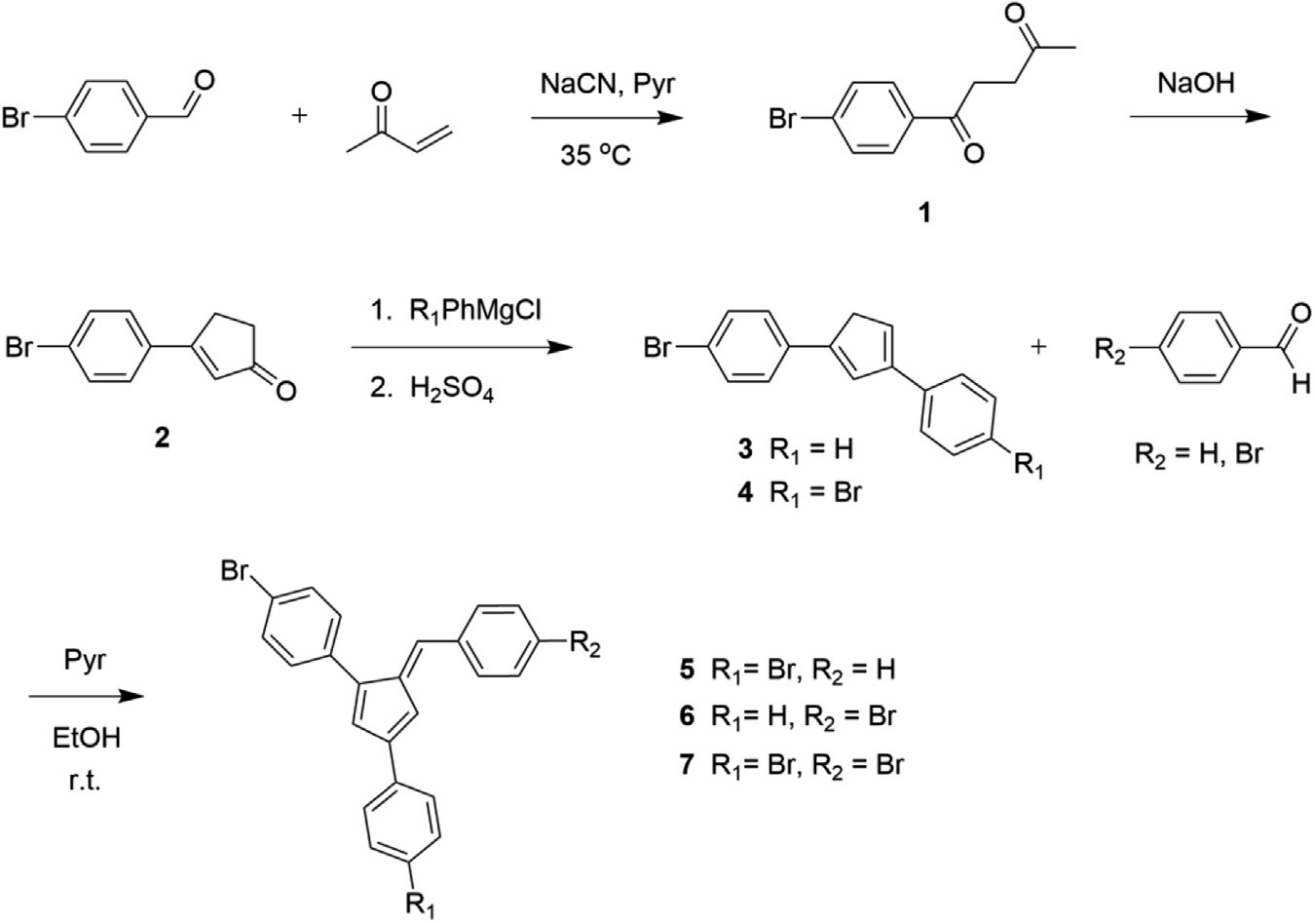
Synthetic scheme for bromine-functionalized fulvene compounds.

Pyrrolidine (57 mg, 0.07 mL, 0.80 mmol) was added drop wise to a vigorously stirred slurry of 4-bromobenzaldehyde (0.12 g, 0.64 mmol) and **4** (0.20 g, 0.53 mmol) in absolute EtOH (100 mL). The mixture was stirred for 24 h at room temperature. The red solution mixture was concentrated by evaporating the solvent to half the volume, vacuum filtered, washed with cold EtOH, and dried under reduced pressure affording the pure product as a dark red solid (216 mg, 75%). ^1^H NMR (400 MHz, CDCl_3_): *δ* 6.93–6.98 (m, 2H); 7.14 (s, 1H), 7.30–7.33 (m, 2H), 7.46–7.62 (m, 10H); ^13^C NMR (100 MHz, CDCl_3_): *δ* 114.66, 121.47, 122.12, 123.93, 127.58, 128.11, 130.80, 131.61, 131.85, 131.97, 132.06, 133.69, 134.38, 135.55, 137.41, 140.58, 144.31, 145.98; HRMS (ESI): Calcd for [M+H]^+^, 540.8804, Found, 540.8906 (Scheme 1).
