# Supplementary material for: Synthesis of reactive 1,3-diphenyl-6-aryl-substituted fulvene chromophores
Source: Data Brief. 2018 Jun 22;19:1638–42. doi: 10.1016/j.dib.2018.06.026 (PMC6141161; doi:10.1016/j.dib.2018.06.026)
Supplement: Supplementary file 1 — Supplementary material [file mmc1.docx]

# May 18, 2018

Conflict of Interest and Authorship Conformation Form for article DIB-D-18-00637

All authors have participated in (a) conception and design, or analysis and interpretation of the data; (b) drafting the article or revising it critically for important intellectual content; and (c) approval of the final version.

This manuscript has not been submitted to, nor is under review at, another journal or other publishing venue.

The authors have no affiliation with any organization with a direct or indirect financial interest in the subject matter discussed in the manuscript

If there are any additional comments and recommendations, please feel free to contact me. Thank you for considering our contribution for publication.

Sincerely,


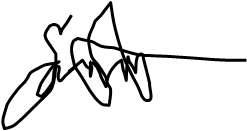


Scott T. Iacono, PhD

Professor, Department of Chemistry

Director, Chemistry Research Center

United States Air Force Academy

Colorado Springs, CO 80840

scott.iacono@usafa.edu
